# Differential Effects of High Methionine Diet on Biochemical Parameters in Normal and Diabetic Rat Models

**DOI:** 10.1055/a-2686-7562

**Published:** 2025-10-29

**Authors:** Yongwei Jiang, Meimei Zhao, Mo Li, HaoYan Zhu, Xiaomu Kong, Qian Liu, Yi Liu, Peng Gao, GuoXiong Deng, Hailing Zhao, Ming Yang, Yongtong Cao, Ping Li, Liang Ma

**Affiliations:** 1 36635 Clinical Laboratory, China-Japan Friendship Hospital, Beijing, China; 2 36635 Beijing Key Lab Immune-Mediated Inflammatory Diseases, Institute of Clinical Medical Science, China-Japan Friendship Hospital, Beijing, China

**Keywords:** methionine metabolism, diabetic complications, hepatic steatosis, diabetic nephropathy, AMPK signaling

## Abstract

This study investigated the organ-specific effects of a high-methionine (HM) diet in streptozotocin (STZ)-induced diabetic rats, focusing on hepatic and renal metabolic adaptations. Male Wistar rats were divided into four groups (n=8/group): normal control, HM (2% methionine), STZ-diabetic, and HM+STZ. Over 12 weeks, HM supplementation in diabetic rats significantly reduced hepatic triglyceride accumulation (42.00±7.71 vs. 20.76±3.63 mg/g tissue, P<0.01), coinciding with AMP-activated protein kinase (AMPK) activation (1.96-fold, P<0.05) and downregulation of lipogenic genes (sterol regulatory element-binding protein 1c ↓63.2%, P<0.05). Conversely, HM exacerbated diabetic nephropathy, elevating urinary albumin-creatinine ratio (411.90±88.86 vs. 238.41±62.52 mg/g, P<0.05) and glomerulosclerosis index (2.5±0.5 vs. 1.8±0.4, P<0.001). Hyperhomocysteinemia (105.69±33.81 μmol/L) persisted across HM groups without altering folate/vitamin B12 levels (P>0.05). These findings demonstrate a striking dichotomy: HM diet ameliorates hepatic steatosis through AMPK-mediated lipid modulation while accelerating renal injury via homocysteine-dependent pathways. The results highlight the need for organ-specific nutritional strategies in diabetes management.

## Introduction

Methionine, a sulfur-containing amino acid, plays a pivotal role in protein synthesis, methylation reactions, and one-carbon metabolism. Emerging evidence suggests methionine metabolism exhibits striking organ-specificity: in the liver, it serves as a glutathione precursor conferring antioxidant protection 1 , while in the kidney, hyperhomocysteinemia (HHcy) exacerbates fibrosis 2 . This dichotomy remains unexplored in diabetes, where both hepatic steatosis and nephropathy commonly coexist 3 .

Notably, hepatic lipid metabolism in diabetes is regulated by sterol regulatory element-binding protein 1c (SREBP-1c), a master transcriptional regulator of lipogenesis that is sensitive to cellular redox status 4 . This intersection between methionine metabolism and diabetic steatosis pathways remains poorly understood. While previous studies have examined high methionine (HM) diet effects in healthy models 5 6 , research under diabetic conditions has been limited. Importantly, emerging evidence suggests that methionine restriction activates adenosine monophosphate-activated protein kinase (AMPK) 7 , but whether HM diets exert similar metabolic regulation remains unknown.

Building upon prior research, this study innovatively established a diabetic rat model with concurrent HHcy to investigate dual metabolic disturbances. We hypothesized that the HM diet would exert divergent organ-specific effects in diabetic rats—ameliorating hepatic dysfunction through AMPK-independent lipid modulation while exacerbating renal injury via HHcy-induced podocyte autophagy impairment 8 9 . By systematically analyzing these effects in both normal and diabetic rats, this study provides novel insights into the complex interplay between methionine metabolism and diabetic complications, offering critical foundations for developing personalized nutritional interventions for diabetic patients.

## Materials and Methods

### Animal model establishment

This study was conducted following approval from the Animal Ethics Committee of the China-Japan Friendship Hospital (Approval No. Zryhyy12-20-01-09) and strictly followed the ARRIVE guidelines for animal management and experimental procedures 10 . Male 8-week-old Wistar rats (weighing 250–300 g) were obtained from Beijing Vital River Laboratory Animal Technology Co., Ltd. (Beijing, China). Animals were acclimatized to the laboratory environment for one week before the start of the experiments. To minimize bias, all animals were randomly assigned to four groups (n=8 per group) as follows:

1. Normal control group: Rats were fed a standard diet for 12 weeks and served as the control group.
2. Hyper-methionine group (HM Group): Rats were fed a diet enriched with 2% methionine 11 for 12 weeks to assess the effects of HM on healthy rats.
3. Streptozotocin (STZ)-induced diabetic group (STZ Group): Diabetes was induced by intraperitoneal injection of STZ at a dose of 30 mg/kg, administered twice within one week. STZ was freshly prepared in 0.1 mol/L citrate buffer (pH 4.5). Rats with blood glucose levels exceeding 16.7 mmol/L in the caudal vein for 3 consecutive days after the second STZ injection were confirmed as diabetic and included in the study 12 . This group received a standard diet for 12 weeks.
4. HM+STZ Group: Diabetes was induced using the same STZ protocol as in the STZ group. After confirmation of hyperglycemia, rats in this group were fed a 2% methionine-enriched diet for 12 weeks to assess the effects of HM in diabetic conditions.

Blood glucose levels, liver function markers, and kidney function indicators were measured before treatment (baseline) and every 4 weeks after treatment across all groups. Body weights were measured at baseline (prior to intervention) and at the experimental endpoint.

### Homocysteine (Hcy)-related indicators

Plasma levels of Hcy and its related metabolic indicators, including folic acid (FA) and vitamin B12 (vit B12), were measured using a Roche automated biochemical analyzer following the manufacturer’s instructions.

### Blood glucose, blood lipid profile, and liver function determination

The levels of fasting blood glucose (FBG), triglycerides (TG), total cholesterol (TC), high-density lipoprotein cholesterol (HDL-c), and low-density lipoprotein cholesterol (LDL-c), as well as alanine aminotransferase (ALT), aspartate aminotransferase (AST), total protein (TP), albumin (ALB), and A/G (ALB/GLB) in rat plasma were measured using a Roche biochemical analyzer according to the manufacturer’s instructions.

### Biochemical assays for liver triglyceride content and redox status

After 12 weeks of treatment, the left lobe of the liver was excised, rinsed in ice-cold phosphate buffered saline (PBS), snap-frozen in liquid nitrogen, and stored at−80°C until analysis. Tissues were homogenized (1:9 w/v) in cold PBS (pH 7.4) using a high-speed tissue homogenizer KZ-III-F (Servicebio) followed by centrifugation at 10,000×g for 15 min at 4°C. The supernatant was collected for assays. Liver TG content was measured using a Roche biochemical analyzer according to the manufacturer’s instructions. Measurement of MDA, T-SOD, and glutathione (GSH) in liver tissue using malondialdehyde (MDA) Assay Kit (for tissue and blood Samples, Servicebio, G4302), Total Superoxide Dismutase (T-SOD) Assay Kit (Servicebio, G4306), and Reduced Glutathione (GSH) Assay Kit (Servicebio, G4305), respectively.

### RNA extraction and quantitative polymerase chain reaction (qPCR) analysis​

Total RNA was extracted from rat liver tissues using TRIzol reagent (Invitrogen) according to the manufacturer's protocol. RNA purity and concentration were determined spectrophotometrically using Nanodrop 2000 (Thermo Scientific). First-strand cDNA was synthesized from 1 μg total RNA using the SweScript All-in-One RT SuperMix for qPCR (OneStep gDNA Remover, Servicebio). Quantitative real-time PCR was performed on a Gentier 96 system (Tianlong) with SYBR Green Master Mix (Servicebio), using the following primer sets:

SREBP-1c (F:5'- CGCTCTTGACCGACATCGA,

R:5'- GGCACGGACGGGTACATCTT)

ACC (F:5'- ACATCCCGCACCTTCTTCTACT,

R:5'- CCACAAACCAGCGTCTCAAC)

CPT1A (F:5'- GAGTGCCAGGAGGTCATAGATGC,

R:5'- CAGTCTCTGTCCTCCCTTCTCG)

Glyceraldehyde-3-phosphate dehydrogenase (GAPDH) served as the internal control. Relative mRNA expression was calculated via the 2^ ^(-ΔΔCt)^ method.

### Protein extraction and western blotting

Liver tissues were homogenized in RIPA buffer containing protease/phosphatase inhibitors (Pierce). Protein concentrations were determined by BCA assay (Beyotime). Equal amounts (30 μg) of protein lysates were separated by 10% sodium dodecyl sulfate-polyacrylamide gel electrophoresis and transferred to polyvinylidene fluoride membranes. After blocking with 5% non-fat milk, membranes were incubated overnight at 4°C with primary antibodies against:

Phospho-AMPK (Servicebio, GB114323), total AMPK (Affinity, AF6423), SREBP-1c (Servicebio, GB11524), 1-aminocyclopropane-1-carboxylic acid (ACC, (Affinity, AF6421), carnitine palmitoyltransferase 1A (CPT1A, Affinity, DF12004), GAPDH (Servicebio, GB15004). Horseradish peroxidase-conjugated secondary antibodies (Servicebio, GB23303) were applied for 1 h at room temperature. Protein bands were visualized using enhanced chemiluminescence substrate (Millipore) and quantified by AIWBwell (Servicebio). Phospho-AMPK signals were normalized to total AMPK, and other proteins were normalized to GAPDH.

### Renal function detection

At baseline (week 0) and at weeks 4, 8, and 12, rats were placed in metabolic cages for 24-h urine collection. Urine albumin and creatinine levels were measured using a Roche biochemical analyzer according to the manufacturer’s guidelines. The 24-h urine albumin/creatinine ratio was then calculated.

### Histopathological stains

To evaluate renal morphological changes, kidney tissues from each group were collected after 12 weeks of treatment. The kidney samples were fixed in 10% formalin for 48 h and then embedded in paraffin. Serial sections (3 μm thick) were prepared and stained with hematoxylin and eosin, periodic acid-Schiff (PAS), and Masson’s trichrome stain for histopathological examination.

### Glomerulosclerosis quantification

The glomerulosclerosis index (GSI) was calculated using a modified Raij semi-quantitative method. Each glomerulus was graded 0–3 based on the percentage of sclerotic area: grade 0 (<1%), 1 (1–25%), 2 (26–50%), or 3 (>50%), with additional consideration of pathological features including mesangial expansion, capillary loss, and crescent formation. The GSI was derived from the formula: (∑(Grade_i×Area_i)/Total glomerular area)×100. All analyses were performed using ImageJ with standardized thresholding for PAS-positive matrix quantification (MA/TA ratio). For quality control, 20% of samples underwent blinded re-evaluation by two renal pathologists (κ>0.85), with discordant cases resolved through 3D deconvolution analysis (DeconvolutionLab2 plugin).

### Statistical analysis

All statistical analyses were performed using GraphPad Prism 8.0. Data are presented as mean±standard deviation (SD) for parametric data and median (interquartile range) for non-parametric data in text and tables (n=8/group). Statistical significance was set at p<0.05 (two-tailed). The analysis focused on three primary comparisons: (a) HM vs. normal groups, (b) HM+STZ vs. STZ groups, and (c) HM vs. For cross-sectional parametric data with equal variances, we used two-tailed Student’s t-tests, applying Welch’s correction when variances were unequal (as confirmed by the F-test). Multiple group comparisons were performed using one-way ANOVA (analysis of variance) with Tukey’s post-hoc test when appropriate.

## Results

### High methionine diet induces homocysteine expression in Wistar rats

**Fig. 1:**
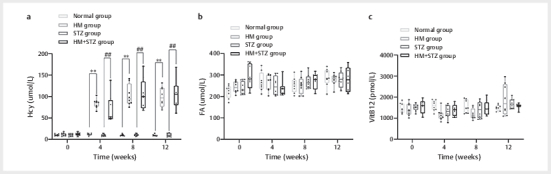
The HM diet induces HHcy in Wistar rats. Rats were fed either a normal chow diet (normal group), an HM diet (HM group), or were STZ-induced diabetic rats fed either a normal chow (STZ group) or an HM diet (HM+STZ group) over 12 weeks. ( **a** ) Plasma homocysteine levels; ( **b** ) Longitudinal changes in folic acid profiles; ( **c** ) Variation in vitamin B12 levels. Data are presented as mean±standard deviation (n=8 per group). ** P<0.01 indicates a statistically significant difference compared with the control (normal group). ## P<0.01 indicates a statistically significant difference compared with the control group (normal chow). HM: high methionine; HHcy: hyperhomocysteinemia; STZ: streptozotocin; Hcy: Homocysteine.

To assess the impact of an HM diet on Hcy levels, Wistar rats were fed either a standard diet or an HM diet for 12 weeks. Compared with the control group, rats in the HM and HM+STZ groups exhibited a significant increase in plasma Hcy levels from week 4 of HM feeding. In the HM group, Hcy levels reached 84.04±11.29 μmol/L, 102.61±26.04 μmol/L, and 99.08±22.82 μmol/L at weeks 4, 8, and 12, respectively, which were significantly higher than those observed in the control group (11.75±2.43 μmol/L, 10.13±1.94 μmol/L, and 9.77±2.11 μmol/L; P<0.01). Similarly, in the HM+STZ group, Hcy levels were 66.38±30.66 μmol/L, 104.99±36.43 μmol/L, and 105.69±33.81 μmol/L at the same time points, which were significantly higher than those in the STZ group (10.22±2.54 μmol/L, 11.20±2.31 μmol/L, and 9.59±2.21 μmol/L; P<0.01), but not significantly different from the HM group (P>0.05, Fig.1a ).

Notably, serum FA ( Fig. 1b ) and vitamin B12 ( Fig. 1c ) levels remained unchanged across all groups throughout the intervention period (P>0.05).

Consistent with the metabolic changes, body weight measurements revealed distinct patterns across groups ( **Supplementary Table 1** ). While normal and HM-fed rats showed expected weight gain (+28.1% and+25.1% respectively), STZ-induced diabetic animals exhibited significant weight loss (−5.3%) that was not ameliorated by feeding on HM (−3.4%, p=0.32 vs. STZ).

### Effect of high methionine on blood glucose, blood lipid profile, and liver function

**Fig. 2:**
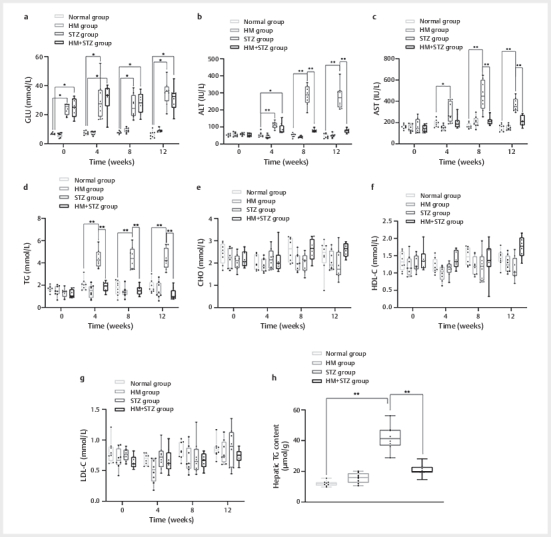
Effects of the HM diet on blood glucose, lipid profile, and liver function in Wistar rats. Rats were fed either a normal chow diet or an HM diet, with or without STZ-induced diabetes, for 12 weeks. ( **a** ) Plasma glucose concentrations, ( **b** ) ALT levels, ( **c** ) AST levels, ( **d** ) TG levels, ( **e** ) CHO levels, ( **f** ) HDL-C levels, and ( **g** ) LDL-C levels. Data are presented as mean±standard deviation; ( **h** ); Hepatic TG content. (n=8 per group). *P<0.05 indicates a statistical difference and **P<0.01 indicates a significant difference. HM: high methionine; HHcy: hyperhomocysteinemia; STZ: streptozotocin; ALT: alanine aminotransferase; AST: aspartate aminotransferase; TG: triglyceride; CHO: total cholesterol; HDL-C: high-density lipoprotein cholesterol; LDL-C: low-density lipoprotein cholesterol; GLU: glucose.

Feeding rats with a 2% methionine-enriched diet did not significantly affect blood glucose levels in the HM group (P>0.05, Fig. 2a ). In the STZ group, blood glucose levels were significantly elevated at all time points (week 0: 24.09±3.88 mmol/L, week 4: 30.01±12.73 mmol/L, week 8: 25.78±8.14 mmol/L, and week 12: 35.18±8.48 mmol/L) compared with the normal group (P<0.05). Similarly, in the HM+STZ group, blood glucose levels were 24.84±6.89 mmol/L, 31.29±9.55 mmol/L, 27.96±7.19 mmol/L, and 30.98±8.33 mmol/L at weeks 0, 4, 8, and 12, respectively, which were also significantly higher than those in the normal group (P<0.05, Fig. 2a ). Consistent with the HM group, there was no statistically difference in blood glucose levels between the STZ and HM+STZ groups (P>0.05, Fig. 2a ) which indicates that HM diet has no significant effect on blood glucose levels in either normal or diabetic Wistar rats.

We observed intriguing results regarding ALT, AST, and TG levels. ALT levels in the HM group did not show significant differences compared with the control group at any time point (P>0.05). In contrast, the STZ group exhibited significantly elevated ALT levels at weeks 4, 8 and 12, measuring 111.00±20.13 IU/L, 285.13±58.63 IU/L, and 277.38±69.59 IU/L, respectively, compared with the normal group (55.13±13.78 IU/L, 51.88±10.78 IU/L, and 47.25±12.70 IU/L, P<0.01, Fig. 2b ). Interestingly, in HM-fed STZ rats (HM+STZ group), ALT levels were markedly reduced at weeks 4, 8, and 12 (88.38±31.67 IU/L, 78.63±9.30 IU/L, and 77.50±12.29 IU/L respectively) compared with the STZ group (P<0.01, Fig. 2b ).

Similarly, AST levels in the HM group did not differ significantly from the normal control group (P>0.05). The STZ group exhibited markedly increased AST levels at weeks 4,8, and 12 (295.50±86.46 IU/L, 458.13±133.21 IU/L, and 366.50±62.91 IU/L) compared with the normal group (192.00±32.29 IU/L; P<0.05; 167.5±24.28 IU/L and 154.75±30.26 IU/L; P<0.01). In contrast, HM-fed STZ rats (HM+STZ group) demonstrated significantly reduced AST levels (209.63±54.89 IU/L, 219.14±34.99 IU/L, and 218.86±51.47 IU/L; P<0.01), which were comparable to those of the normal group (P>0.05, Fig. 2c ).

As comprehensively summarized in **Supplementary Table 2** , STZ-induced diabetic rats exhibited significant elevations in both ALT and AST levels compared with normal controls (both p<0.01), whereas HM co-administration markedly attenuated these changes. Lipid profile analysis demonstrated that from week 4 to week 12 of feeding, the STZ group had significantly higher TG levels (4.58±0.80 mmol/L, 4.24±1.01 mmol/L, and 4.34±0.91 mmol/L) than the normal group (2.08±0.52 mmol/L, 1.74±0.65 mmol/L, and 1.86±0.58 mmol/L; P<0.01). Intriguingly, the HM+STZ group exhibited significantly lower TG levels (1.20±0.36 mmol/L, 1.88±0.43 mmol/L, and 1.56±0.45 mmol/L) than the STZ group (P<0.01), returning to normal levels (P>0.05, Fig. 2d ). No statistically significant differences were observed in TC, HDL-C, or LDL-C levels among the groups ( Fig. 2e–g ).

To further assess the impact of the HM diet on hepatic lipid metabolism, we quantified TG content in liver tissues. Consistent with the plasma TG reduction ( Fig. 2d ), the HM+STZ group exhibited an obvious decrease in hepatic TG level compared with that in the STZ group (20.76±3.63 vs. 42.00±7.71 mg/g tissue; P<0.01; Fig. 2h ). In contrast, the HM group showed no difference from normal controls (15.68±3.11 vs. 12.13±1.67 mg/g tissue; P>0.05). This reduction in hepatic TG was aligned with the amelioration of ALT/AST elevations ( Fig. 2b, c ), suggesting that the HM diet may alleviate STZ-induced hepatic steatosis and subsequent injury.

### High methionine potentiates streptozotocin-induced hepatic oxidative damage via the AMPK pathway suppression

Quantitative analysis revealed STZ-induced hepatic oxidative stress, evidenced by 202.46% higher MDA (7.38±1.00 vs. 2.44±0.33 μmol/g prot, p<0.01), 44.48% lower GSH (5.13±1.65 vs. 9.24±1.32 μmol/g prot, p<0.01), and 52.37% reduced T-SOD (97.33±24.42 vs. 204.37±24.01 U/mg prot, p<0.01) in STZ vs. Normal groups ( Fig. 2a–c ). HM co-administration (12 weeks) potentiated these effects, yielding a 41.60% reduction in MDA (4.31±0.66 μmol/g prot, p<0.01 vs. STZ group), 71.73% GSH elevation (8.81±2.20 μmol/g prot, p<0.01 vs. STZ group ), and 89.84% T-SOD activity (184.75±39.83 U/mg prot, p<0.01 vs. STZ group) recovery.

**Fig. 3:**
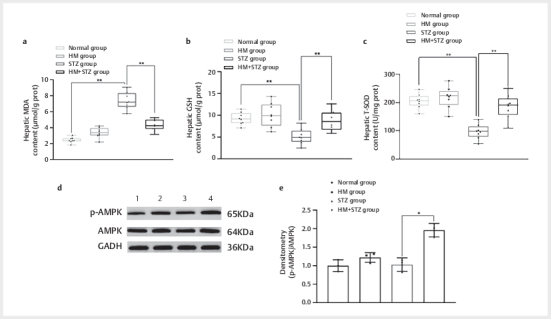
Hepatic metabolic profiling and AMPK pathway activation in experimental rat groups. ( **a** ) Hepatic MDA content; ( **b** ) Hepatic GSH content; ( **c** ) Hepatic T-SOD content; ( **d** ) Western blot analysis of p-AMPK(65 KDa), AMPK (64 KDa), and GAPDH (36 KDa, loading control) expression in rat liver tissues under varying conditions (lanes 1–4); ( **e** ) Densitometry of p-AMPK/AMPK. Lane 1: normal group; Lane 2: HM group; Lane 3: STZ group; Lane 4: HM+STZ group. Representative blots are shown. *P<0.05 indicates a statistical difference and **P<0.01 indicates a significant difference. HM: high methionine; AMPK: AMP- activated protein kinase; p-AMPK: phosphorylated AMPK; MDA: malondialdehyde; GSH: glutathione; T-SOD: total superoxide dismutase; GADH: glyceraldehyde-3-phosphate dehydrogenase.

To explore the potential mechanism underlying the HM diet’s hepatoprotective effects, we analyzed hepatic AMPK activation (phospho-AMPK/AMPK ratio) by Western blot. The HM+STZ group exhibited a significant increase in p-AMPK/AMPK levels compared with the STZ group (1.96±0.15 vs. 1.03±0.15, P<0.05; Fig. 3d, e ), whereas no difference was observed between the HM and normal control groups (P>0.05). This AMPK activation coincided with the amelioration of ALT/AST elevations ( Fig. 2b, c ) and hepatic TG accumulation ( Fig. 2h ), suggesting that the HM diet may mitigate STZ-induced liver injury through AMPK-dependent pathways. Notably, despite AMPK activation, blood glucose levels remained unchanged in HM+STZ rats ( Fig. 2a ), indicating a selective metabolic modulation.

### High methionine diet modulates hepatic lipid metabolism pathways in diabetic rats

**Fig. 4:**
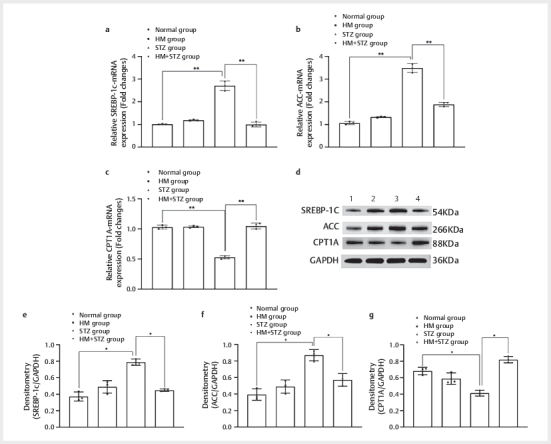
Hepatic lipogenic gene expression and protein levels in experimental groups. (A) qPCR quantification of ( **a** ) Srebp-1c, ( **b** ) Acc, and ( **c** ) Cpt1a genes. Data normalized to GAPDH and expressed as fold-change (mean±SEM, n=8). ( **d** ) Western blot analysis of SREBP-1C (54 kDa), ACC (266 kDa), CPT1A (88 kDa) and GAPDH (36 kDa, loading control). Lanes: 1–4 represent biological replicates per group: Lane 1: Normal group; Lane 2: HM group; Lane 3: STZ group; Lane 4: HM+STZ group. ( **e** ) Densitometry of SREBP-1C /GAPDH; ( **f** ) Densitometry of ACC /GAPDH; ( **g** ) Densitometry of CPT1A /GAPDH. *P<0.05 indicates a statistical difference and **P<0.01 indicates a significant difference. qPCR: quantitative polymerase chain reaction; SREBP-1c: sterol regulatory element-binding protein 1c; ACC: 1-aminocyclopropane-1-carboxylic acid; CPT1A: carnitine palmitoyltransferase 1A; GAPDH: glyceraldehyde-3-phosphate dehydrogenase; SEM: standard error of the mean; HM: high methionine; STZ: streptozotocin.

Western blot and qPCR analyses revealed coordinated yet distinct regulatory patterns of hepatic lipid metabolism mediators under HM intervention ( Fig. 4 ). Quantitative PCR of liver tissues (n=8/group) demonstrated that STZ-induced diabetes significantly upregulated SREBP-1C mRNA expression (2.71±0.17-fold vs. normal controls, P<0.01, one-way ANOVA with Tukey’s test), which was attenuated by 63.16% in HM+STZ group (1.00±0.05 vs. STZ, P<0.05), suggesting suppression of lipogenesis ( Fig. 4a ). ACC mRNA levels in STZ group increased by 227.80% (3.49±0.19, P<0.01 vs. normal), consistent with hyperlipidemia, whereas the level was downregulated by 46.05% in HM+STZ group (1.89±0.08, P<0.01 vs. STZ), indicating reduced malonyl-CoA production and enhanced fatty acid oxidation ( Fig. 4b ). The level of CPT1A remained stable across groups (HM: 1.03±0.01, P>0.05 vs. normal), but was significantly suppressed in STZ rats (0.53±0.02 vs. normal, P<0.01) with a significant recovery trend in HM+STZ animals (1.05±0.04 vs. STZ, P<0.01, Fig. 4c ).

At the protein level, western blot analysis (normalized to GAPDH) showed parallel changes for SREBP-1C (54 kDa), with the STZ group exhibiting a 2.12-fold increase versus normal (P<0.001) and the HM+STZ group showing a 43.09% reduction (P<0.01, Fig. 4d, e ). The level of ACC (266 kDa) protein increased 2.21-fold in the STZ versus the normal group (P<0.01, Fig. 4d–f ). The expression of CPT1A protein (88 kDa) was suppressed in the STZ group (0.41±0.03 vs. normal, P<0.01) with HM intervention eliciting 98.68% recovery at the protein level (P<0.01 vs. STZ, Fig. 4d–g ).

### Effect of high methionine on renal function and glomerular morphology

**Fig. 5:**
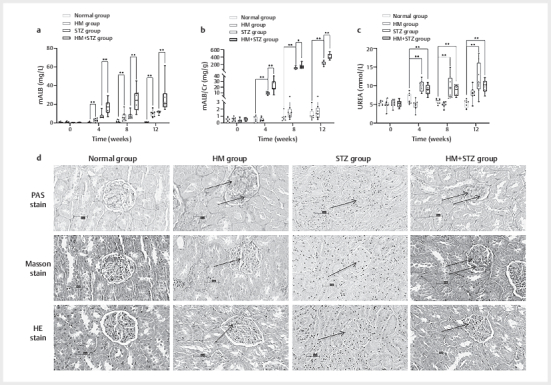
Effects of an HM diet on renal function and glomerular morphology in Wistar rats. Rats were fed either a normal chow diet or an HM diet, with or without STZ-induced diabetes, for 12 weeks. ( **a** ) mALB levels; ( **b** ) mALB/creatinine ratio (Cr); ( **c** ) Blood urea levels. Data are expressed as mean±standard deviation (n=8 per group). ( **d** ) Representative images of renal tissue stained with PAS, Masson, and HE after 12 weeks (×400 magnification). *P<0.05 indicates a statistical difference compared with the control (normal group). HM: high methionine; STZ: streptozotocin; mALB: 24-hour urinary albumin; PAS: periodic acid-Schiff; HE: hematoxylin and eosin.

Proteinuria assessment revealed that the HM group exhibited significantly higher 24-hour urinary microalbumin levels at weeks 4, 8, and 12 (2.73±1.84 mg/L, 7.29±4.45 mg/L, and 9.89±3.67 mg/L) compared with the normal group (0.70±0.67 mg/L, 1.74±1.39 mg/L, and 1.74±1.39 mg/L; P<0.05). Notably, the HM+STZ group demonstrated substantially elevated 24-hour urinary microalbuminlevels (17.12±8.44 mg/L, 24.37±14.09 mg/L, and 26.59±17.63 mg/L) compared with the STZ group (7.76±3.92 mg/L, 7.92±4.23 mg/L, and 11.66±1.88 mg/L; P<0.05; Fig. 5a ).

During the 12-week HM dietary intervention, no significant difference in urinary albumin-to-creatinine ratio (UACR) was observed between the HM group and normal controls (P>0.05). In striking contrast, both STZ and HM+STZ groups showed progressive elevation of UACR from week 4 onward (P<0.05). The STZ group exhibited 24-hour urinary microalbuminvalues of 9.30±2.92 mg/g, 108.88±58.70 mg/g, and 238.41±62.52 mg/g at weeks 4, 8, and 12 respectively, while the HM+STZ group displayed significantly higher values (21.37±12.13 mg/g, 127.30±81.33 mg/g, and 411.90±88.86 mg/g; P<0.05 vs. STZ group from week 4; Fig. 5b ). These findings demonstrate that HM diet significantly exacerbates the increase in UACR in diabetic rats, indicating accelerated renal functional impairment compared with that in standard diet-fed STZ diabetic rats.

Blood urea analysis showed that both STZ (10.18±1.96 mmol/L, 11.72±5.01 mmol/L, and 11.88±4.29 mmol/L) and HM+STZ groups (9.24±1.94 mmol/L, 8.69±1.99 mmol/L, and 9.95±2.35 mmol/L) had significantly elevated levels compared with normal controls from week 4 (P<0.05; Fig. 5c ). However, no significant differences were observed in 24-hour urinary urea excretion between HM and normal groups, or between STZ and HM+STZ groups, suggesting that HM diet does not significantly alter the rate of urea excretion in diabetic rats.

Histopathological examination revealed that both STZ and HM+STZ groups developed more severe glomerulosclerotic lesions compared with normal controls, with prominent mesangial matrix expansion. The HM+STZ group exhibited more advanced pathological changes than the STZ group. Importantly, the HM group maintained normal glomerular morphology comparable to controls ( Fig. 5d ), consistent with the functional measurements.

Quantitative assessment of glomerulosclerosis revealed significant differences among the four experimental groups ( Table 1 ). Normal control rats exhibited minimal glomerular injury, with a mean glomerulosclerosis index (GSI) of 0.02±0.01 and 98% of glomeruli classified as Grade 0 (no sclerosis). In contrast, the HM diet group demonstrated mild glomerular damage (GSI: 1.1±0.3*, p<0.05 vs. normal), characterized predominantly by focal mesangial expansion (Grade 1: 30% of glomeruli). STZ-induced diabetic rats developed more severe glomerulosclerosis (GSI: 1.8±0.4, p<0.01 vs. HM group), with 30% of glomeruli showing Grade 2 lesions (26–50% sclerosis with capillary loss). The combined HM+STZ group exhibited the most pronounced renal pathology (GSI: 2.5±0.5, p<0.001 vs. STZ alone), including a high prevalence of global sclerosis (Grade 3: 50% of glomeruli) and crescent formation.

**Table:** **Table 1** Comparison of Glomerulosclerosis Index (GSI) across experimental groups.

Group	Mean GSI±SEM	Proportion of Grade 0	Proportion Grade 1	Proportion Grade 2	Proportion Grade 3
Normal group	0.02±0.01	98%	2%	0%	0%
HM group	1.1±0.3*	65%	30%	5%	0%
STZ group	1.8±0.4**​	20%	45%	30%	5%
HM+STZ group	2.5±0.5*​**​	5%	25%	40%	30%

* vs. normal group, p<0.05,** vs. normal group, p<0.01,*​** vs. STZ group p<0.001 (ANOVA+Tukey’s test); HM: high methionine; STZ: streptozotocin.

## Discussion

This study focused on hepatic and renal responses to the HM diet in diabetic rats, employing functional biomarkers, histological evaluation (kidney), and molecular analyses (liver) to elucidate organ-specific metabolic adaptations. Our findings reveal a striking dichotomy in how an HM diet affects diabetic complications, demonstrating significant hepatic protection alongside exacerbated renal injury. This organ-specific response occurred despite comparable induction of hyperhomocysteinemia (HHcy) across groups (84.04–105.69 μmol/L), suggesting distinct tissue-dependent metabolic adaptations to methionine excess. Such organ-specific effects of nutritional interventions are increasingly recognized in metabolic diseases 13 , but the underlying mechanisms remain poorly understood.

The hepatoprotective effects observed in our study were particularly remarkable, with the HM diet attenuating STZ-induced ALT elevations by 72.40% and reducing hepatic TG accumulation by 50.57%. Although the HM diet reduced AST levels by 40.28% (366.50→218.86 IU/L), values remained elevated compared with controls, suggesting residual hepatic injury despite metabolic improvement. Mechanistically, these beneficial effects appear to be mediated through two principal pathways: (1) transcriptional suppression of key lipogenic regulators SREBP-1c and ACC (reduced by 63.16% and 46.05% respectively), with concomitant but insufficient AMPK activation (1.96-fold, P<0.05); and (2) enhanced antioxidant capacity as evidenced by a 71.73% increase in GSH levels (P<0.01, Fig 3b ). These findings are consistent with recent studies demonstrating the redox sensitivity of SREBP-1c in diabetic liver 14 and the crucial role of methionine as a precursor for glutathione synthesis 15 . Notably, studies in UCP-1-deficient models suggest that methionine restriction may exert metabolic benefits through UCP-1-dependent mechanisms 16 , indicating potential alternative pathways beyond classical AMPK activation.

In striking contrast, the HM diet exacerbated diabetic nephropathy, increasing UACR by 72.80% and accelerating glomerulosclerosis progression. This renal vulnerability likely results from multiple synergistic pathological processes: HHcy-induced impairment of podocyte autophagy 17 , transforming growth factor-β/Smad3-mediated potentiation of fibrotic pathways 18 , and insufficient compensatory antioxidant responses compared to the liver. Cutting-edge single-cell RNA sequencing studies have identified renal tubular epithelial cells as being particularly susceptible to homocysteine toxicity due to their limited capacity for homocysteine remethylation 19 . Furthermore, the diabetic kidney demonstrates impaired activity of the transsulfuration pathway 20 , which may exacerbate HHcy-induced oxidative damage 21 . These findings significantly extend previous clinical observations linking HHcy with accelerated nephropathy progression 22 , providing robust experimental evidence for a causal relationship.

Our results may carry clinical implications for dietary considerations in diabetic patients. For individuals with hepatic steatosis but preserved renal function (eGFR>60 mL/min), moderate methionine intake may confer metabolic benefits, as supported by recent nutritional intervention studies 23 . Conversely, patients with established nephropathy (UACR>30 mg/g) should likely maintain stricter dietary methionine restriction, particularly in light of emerging evidence that even mild HHcy accelerates renal function decline in diabetes 24 . This personalized nutritional approach represents a significant advancement beyond current standardized dietary guidelines 25 and aligns with the growing recognition of precision nutrition in diabetes management 26 .

Several important limitations must be considered when interpreting these findings. First, the exclusive use of male animals precludes evaluation of potential sex differences in methionine metabolism, which have been well-documented in both preclinical models 27 and human studies 28 . Second, the 12-week study duration may not fully capture long-term renal outcomes, especially considering evidence that the detrimental effects of HHcy on kidney function may progress over extended periods 29 . Third, while we have identified compelling associations between HM diet and organ-specific effects, establishing definitive causal relationships will require further investigation using tissue-specific knockout models 30 or targeted pharmacological interventions 31 . Fourth, our study focused on male rats, precluding evaluation of sex differences. Given that estrogen enhances homocysteine remethylation via betaine-homocysteine methyltransferase 32 , future studies should include ovariectomized females to assess gender-specific responses to high-methionine diets in diabetic conditions.

Clinically, these findings advocate for personalized nutritional strategies: diabetic patients with hepatic steatosis (e. g., ALT>40 IU/L and CAP≥248 dB/m) may benefit from moderate methionine intake, whereas those with microalbuminuria (UACR>30 mg/g) require strict restriction. This aligns with recent American Diabetes Association guidelines emphasizing individualized medical nutrition therapy 33 .

In conclusion, this study provides a comprehensive demonstration of the divergent organ-specific effects of HM diet in diabetes, fundamentally transforming our understanding of the role of dietary methionine in the pathogenesis of diabetic complications. These findings underscore the critical need for organ-specific nutritional strategies and highlight SREBP-1c regulation as a particularly promising therapeutic target for diabetic steatosis, operating through mechanisms independent of classical AMPK pathways. Future research should prioritize: (1) identification of molecular sensors mediating tissue-specific responses to methionine, (2) development of targeted interventions that preserve hepatic benefits while mitigating renal risks, and (3) translation of these findings into clinically applicable dietary guidelines through rigorously designed randomized controlled trials 34 35 .
